# Combined Subcision and Low‐Volume Diluted Calcium Hydroxylapatite for Gluteal Cellulite: A Three‐Patient Case Series

**DOI:** 10.1111/jocd.71070

**Published:** 2026-07-14

**Authors:** Andrea Acevedo, Luis Alberto Parra, Juan Sebastian Rodriguez Cabrales, Valentina Dicker, Lina Velasquez, Andrea Marcela Parra

**Affiliations:** ^1^ Aesthetic Medicine Rosario University Cali Colombia; ^2^ Aesthetics Medicine North University Barranquilla Colombia; ^3^ Aesthetic Medicine – Clinical Research National University of Colombia Bogotá Colombia; ^4^ Aesthetic Medicine Rosario University Bogotá Colombia; ^5^ Dermatologist. Colombian Association of Dermatology Cali Colombia; ^6^ Oculoplastic Surgery – Ophthalmologist North University Barranquilla Colombia

To the Editor,

1

Cellulite, or edematous fibrosclerotic panniculopathy, affects most post‐pubertal women and arises from vertically oriented fibrous septa that tether the dermis, producing the characteristic dimpling [1]. Conventional modalities seldom achieve durable correction, and high‐volume biostimulator protocols remain financially inaccessible to most patients in low‐resource settings. We describe a combined, low‐volume protocol pairing subcision with diluted calcium hydroxylapatite (CaHA; Radiesse, Merz Pharmaceuticals GmbH, Frankfurt, Germany) for gluteal cellulite in three women.

Three female patients (ages 30–37) with gluteal cellulite were treated by a single practitioner. Focal retractions first underwent subcision with a 22G 50‐mm blunt cannula to release the fibrotic septa, followed by subdermal CaHA at a 1:3 dilution (fanning technique) and focal 1:1 injections through a 27G needle for volumization. Total product was limited to one vial per buttock. Cellulite severity was scored with the Cellulite Severity Scale (CSS) by six blinded specialists (> 8 years' experience) trained on 20 reference images, with discrepancies adjudicated by a third evaluator. Follow‐up was at 3 months (Patients 1 and 3) and 12 months (Patient 2). Written informed consent and ethics approval were obtained.

All three patients improved. Median CSS fell from 12 to 3, a median 71% reduction (range 50%–75%) (Table 1), with consistent reduction in dimpling, smoother skin, and better contour on standardized photographs. The Patient 2 result persisted at 12 months (Figure 1), suggesting sustained remodeling. No bruising, nodules, vascular events, or infection were recorded during early (1–2 week) monitoring or follow‐up.

**TABLE 1:** Individual CSS scores before and after treatment.

Patient	CSS before	CSS after	% reduction
1	6	3	50
2	14	4	71
3	12	3	75
Median [IQR]	12 [9–13]	3 [3–3.5]	71 [50–75]

**FIGURE 1:**
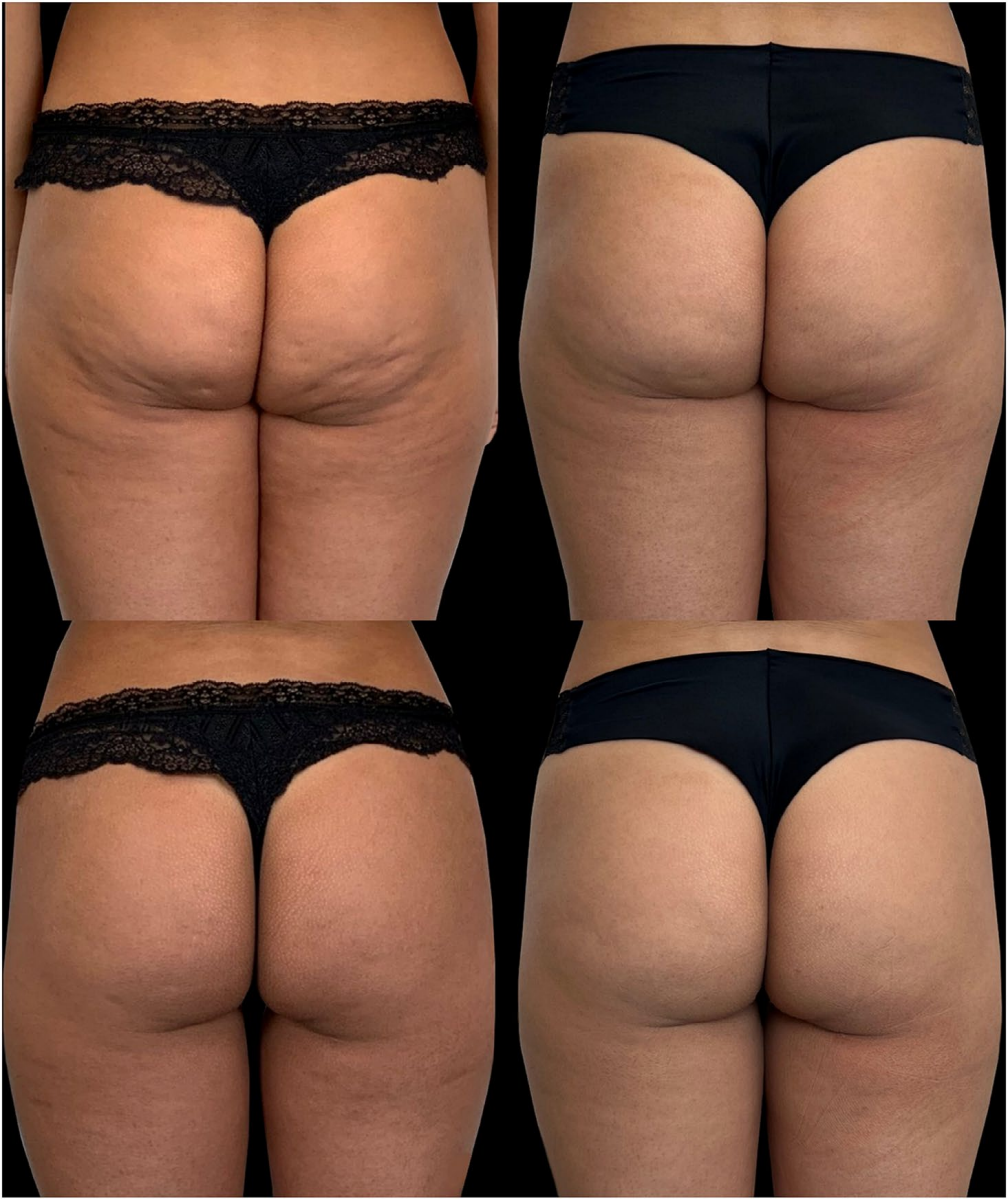
Patient 2, pretreatment (gluteal muscles contracted) and 12‐month follow‐up (relaxed): Reduced dimpling, smoother contour, and sustained volumetric correction.

Subcision mechanically severs the oblique, honeycomb‐like septa that generate cellulite dimples and initiates a wound‐healing response that favors collagen deposition [2]. A single subcision session alone can meaningfully improve CSS [3]. We hypothesized that diluted CaHA, by directly activating fibroblasts within the released septal compartments, could enhance and prolong this neocollagenic response [4]. Our intermediate 1:3 dilution departs from conventional hyperdilution (1:4–1:6): it preserves a microsphere density sufficient for fibroblast engagement while improving spread, balancing biostimulation against dispersion [4]. A recent prospective study of cannula subcision with 1:1 CaHA reported comparable CSS gains, supporting the combined rationale [5].

The protocol's principal advantage is volume economy. Contemporary gluteal protocols advocate 10–20 vials [6], a cost prohibitive for most patients in our setting; achieving meaningful correction with one vial per buttock substantially widens access.

This report is limited by its small, uncontrolled cohort, heterogeneous follow‐up, and—most importantly—the absence of a subcision‐only arm, which prevents isolating the additive effect of CaHA on non‐volumetric outcomes such as skin smoothness. One patient also received prior radiofrequency microneedling, further confounding attribution. Findings should be read as preliminary and hypothesis‐generating.

In conclusion, combined subcision and low‐volume diluted CaHA was well tolerated and associated with clinically meaningful improvement in gluteal cellulite severity in three patients. Its low product requirement makes it an accessible candidate protocol that warrants evaluation against subcision alone in controlled trials.

## Author Contributions

A.A. performed the research. J.S.R.C. designed the research study. A.A. and J.S.R.C. analyzed the data. L.A.P., V.D., L.V. and A.M.P. contributed essential reagents or tools. All authors wrote the paper and have read and approved the final manuscript.

## Ethics Statement

This study was conducted in accordance with the Declaration of Helsinki and was approved by the Ethics Committee for Research on Human Beings HSJ—FUCS (CEISH). The protocol “Retrospective observational clinical study to evaluate the safety and efficacy of injectable medications and medical devices for the improvement of different signs of aging on the body and face” was approved under Act 22 of December 11, 2024 and communication (CEISH) 550–2024.

## Consent

Informed consent was obtained from all subjects involved in the study. The consent process included a detailed explanation of the procedures, potential risks and benefits, and the use of photographic material for documentation and publication purposes.

## Conflicts of Interest

The authors declare no conflicts of interest.

## Data Availability

The data that support the findings of this study are available from the corresponding author upon reasonable request.
